# Enhancing Quality Control: Image-Based Quantification of Carbides and Defect Remediation in Binder Jetting Additive Manufacturing

**DOI:** 10.3390/ma17102174

**Published:** 2024-05-07

**Authors:** Amit Choudhari, James Elder, Manoj Mugale, Sanoj Karki, Satyavan Digole, Stephen Omeike, Tushar Borkar

**Affiliations:** Department of Mechanical Engineering, Washkewicz College of Engineering Cleveland State University, Cleveland, OH 44115, USA

**Keywords:** defects in additive manufacturing, binder jetting, defect analysis, remedies, steel, quality control, carbide quantification, binding mechanisms

## Abstract

While binder jetting (BJ) additive manufacturing (AM) holds considerable promise for industrial applications, defects often compromise part quality. This study addresses these challenges by investigating binding mechanisms and analyzing common defects, proposing tailored solutions to mitigate them. Emphasizing defect identification for effective quality control in BJ-AM, this research offers strategies for in-process rectification and post-process evaluation to elevate part quality. It shows how to successfully process metallic parts with complex geometries while maintaining consistent material properties. Furthermore, the paper explores the microstructure of AISI M2 tool steel, utilizing advanced image processing techniques like digital image analysis and SEM images to evaluate carbide distribution. The results show that M2 tool steel has a high proportion of M_6_C carbides, with furnace-cooled samples ranging from ~2.4% to 7.1% and MC carbides from ~0.4% to 9.4%. M_6_C carbides ranged from ~2.6% to 3.8% in air-cooled samples, while water-cooled samples peaked at ~8.52%. Sintering conditions also affected shrinkage, with furnace-cooled samples showing the lowest rates (1.7 ± 0.4% to 5 ± 0.4%) and water-cooled samples showing the highest (2 ± 0.4% to 14.1 ± 0.4%). The study recommends real-time defect detection systems with autonomous corrective capabilities to improve the quality and performance of BJ-AM components.

## 1. Introduction

**Figure 1:**
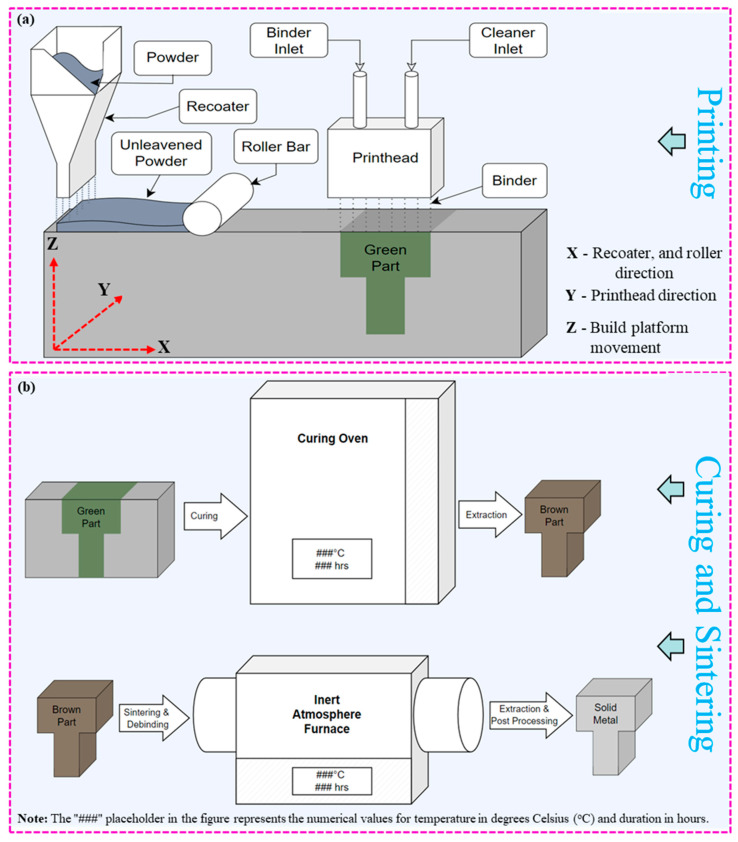
Illustrates the process of BJ, showcasing two key stages: (**a**) the printing phase, where the green part is manufactured using a binder jetting machine, and (**b**) the subsequent steps involving curing the green part in an oven and sintering the brown part in a furnace.

Binder jetting (BJ) additive manufacturing (AM) has emerged as a prominent technology, revolutionizing the manufacturing industry with its ability to produce intricate and complex-shaped components layer by layer. It is an AM process that involves selectively depositing a liquid binding agent onto a powder bed to create three-dimensional objects layer by layer, as shown in Figure 1 [1,2,3,4,5]. Unlike traditional manufacturing methods, BJ offers several unique properties that make it highly versatile and suitable for various industrial applications [6]. One key advantage of BJ is its ability to produce complex geometries with intricate internal features and fine details at atmospheric temperature, hence, no residual stresses [7,8,9]. Additionally, BJ enables the fabrication of parts with a wide range of materials, including metals, ceramics, and polymers, offering flexibility in material selection to meet specific application requirements [10]. The process begins with the deposition of successive layers of powder material onto a build platform, as shown in Figure 1a. A print head then selectively applies a liquid binding agent onto the powder bed, binding the particles together to form each layer of the object. After printing, the green part undergoes a curing process to solidify the binder and strengthen the bond between powder particles, as depicted in Figure 1b. Subsequently, the cured part is subjected to debinding to remove excess binder material. This is followed by sintering in a high-temperature furnace to fuse the powder particles and achieve the final part’s desired properties, as illustrated in Figure 1b. Following the inception of the first 3D printer by Charles W. Hull [11,12,13] in 1984, BJ has garnered significant attention since its invention in 1993 at the Massachusetts Institute of Technology [14,15] due to its versatility in printing various materials and its potential to streamline the production process [16]. However, the realization of BJ’s full potential is hindered by the occurrence of defects during the printing process, which can adversely affect the quality and performance of printed components [17,18,19].

Quality control is paramount in BJ-AM to produce high-quality components with precise dimensional accuracy and mechanical integrity [20,21]. According to statistical data, defects such as warping, deformation, rough surfaces, and low dimensional accuracy are prevalent in BJ printing processes, leading to suboptimal part performance [22,23,24]. For instance, studies have shown that porosity levels ranging from ~2.7% [25] to ~16.4% in BJ-printed components can significantly impact mechanical strength, thermal conductivity, and electrical conductivity [25,26,27]. Understanding the root causes of these defects and implementing effective remediation strategies are essential for enhancing the overall quality of BJ-printed parts. The demand for top-quality parts by the consumer market and the growing use of AM processes to create these parts have led to a greater need for quality control in AM [28,29,30,31]. Satisfying this demand requires a process by which final part quality can be predicted, given knowledge of the starting materials and the AM processes used. With such a model, sources of part defects can be identified and possibly corrected, eliminating the need for costly trial and error [20]. The first step in creating a quality control model is to thoroughly understand the relationships between the process, the starting materials, and the final part quality.

It is imperative to comprehend the potential defects that might arise during the printing process, their underlying causes, their implications for the final product’s performance, and the methodologies to address them [26,32,33,34]. Dini et al. [35] emphasize BJ’s binder injection and sintering processes; they tend to overlook broader defect categories originating from binder accumulation and penetration within the powder bed, such as bleeding and weak bonding. Similarly, Pastre et al. [23] offer an initial review of defects encountered in BJ formation but provide a less comprehensive analysis primarily focusing on macro-scale defects. In another research, Parab et al. [24] provided an enhanced understanding of the fundamental physics governing BJ processes using a high-speed synchrotron X-ray imaging technique, which is now poised to improve the quality of parts produced through BJ. Jian et al. [36] introduced a common defect in BJ known as bleeding, characterized by the macroscopic movement of the binder due to excessive saturation. This phenomenon often results in heavily bound powder accumulating at the base of the printed component. Li et al. [32] explored the impact of a positive rotational motion during powder spreading on surface defects in a compacted powder bed. They noted a recurrent pattern of “surface ridges” forming on the surface, with the number of ridges increasing with each additional layer of powder before reaching a stable point. This observation indicates that the formation of surface ridges is cumulative, potentially leading to other defects like smearing, layer shift, and internal cracks, suggesting over-compaction in the powder bed. Onrel et al. [37] found that weak binding defects result from insufficient strength in the green parts, limiting the ability to print fine features due to structural constraints. Various researchers focused on defects arising from binder saturation levels. Bai et al. [38] demonstrated that if saturation is too low, the curing process in the previous layer leads to partial collapse of the powder spread layer, affecting the pattern of the subsequent layer. On the other hand, Persson et al. [39] found that if saturation is excessively high, it causes the printed part to expand, leading to contact with the powder-spreading mechanism, thereby distorting or damaging the printed pattern. These observations underscore the critical need to control binder saturation levels in BJ to avoid various surface and structural defects.

Existing research on BJ primarily centers on aspects such as printing binders, materials, and process parameters [19,20,40]. However, the interrelation of defects throughout these processes is frequently underestimated, and comprehensive causes and remedies are lacking. Quality control in BJ-AM can be enhanced by systematically studying the possible defects encountered during and after the printing process [35,41]. This research aims to provide a comprehensive overview of defects associated with BJ printing, delving deeply into their mechanisms, relationships, and mitigation strategies. The significance of this research lies in its potential to improve the understanding of defect formation in BJ printing and provide guidance for developing more robust quality control measures.

High-speed steel (HSS) is a category of alloyed steel known for its hardness, wear resistance, and ability to withstand high temperatures during cutting operations. M2 is a specific grade of HSS, prized for its exceptional wear resistance and high hardness retention at elevated temperatures. It is extensively utilized in cutting tools, drills, taps, and various high-speed machining applications [42,43,44,45,46,47]. Quantifying carbides in M2 tool steel processed via BJ is paramount, given its profound implications for material performance, quality assurance, and process optimization within the AM realm. Accurate carbide quantification enables precise control over material characteristics, ensuring that final components meet stringent specifications for hardness, wear resistance, and toughness [48]. Hecht et al. [49] used digital image processing to quantify the networks of carbides for ultrahigh carbon steel. In another study, Kang et al. [50] used the image analysis technique of scanning electron microscopy (SEM) images to characterize the carbides. In this research, the SEM images were used to quantify the different types of carbide networks in the M2 tool steel processed via BJ.

Our research endeavors to delve into various categories of defects encountered in BJ-AM, encompassing both in-process defects detected during printing, such as those arising from powder feeding and powder–binder interaction, and post-process defects identified after printing, including smearing, state cliff face effect, cracks, and layer shift. Recognizing that quality control is pivotal in ensuring the production of high-quality components in BJ-AM, this study aims to analyze and address defects encountered during the printing process systematically. This research aspires to make significant contributions to advancing quality control measures within the realm of BJ-AM, with the goal of fostering its widespread adoption across various industries. Additionally, this research endeavors to quantify the percentage of carbides present in the microstructure of M2 tool steel processed via BJ, further augmenting its contributions to the field.

## 2. Materials and Methods

### 2.1. Powder

**Figure 2:**
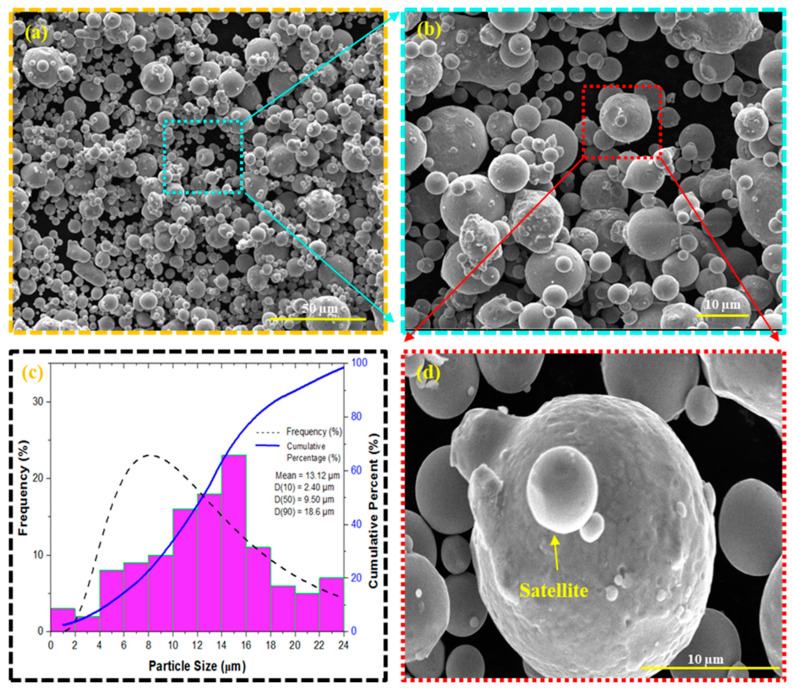
Illustrates scanning electron microscope (SEM) images depicting gas atomized AISI M2 tool steel: (**a**) at a magnification of 100×, (**b**) a further magnified image at 600×, (**c**) the particle size distribution of the powder, and (**d**) the minimal occurrence of satellite particles.

The AISI M2 HSS powder used in BJ experiments was obtained commercially from SANDVIK (Sandvik Osprey Powders, Clarks Summit, PA, USA). Table 1 presents the detailed chemical composition of AISI M2 tool steel. SEM images in Figure 2 illustrate the powder’s predominantly spherical shape, with a minor presence of satellite particles, as shown in Figure 2d. ImageJ software was utilized to measure the powder particle size from scanning electron microscope (SEM) images, as illustrated in Figure 2b. A total of 250 measurements were taken across a range of spherical powders to ensure statistical robustness. The particle size distribution graph is presented in Figure 2c, which clearly shows that the D_10_, D_50_, and D_90_ values of the AISI M2 tool steel powders are 2.40, 9.50, and 18.6 µm, respectively, with a mean size of ~13.12 µm.

### 2.2. Printing, Curing, Debinding, and Sintering

**Figure 3:**
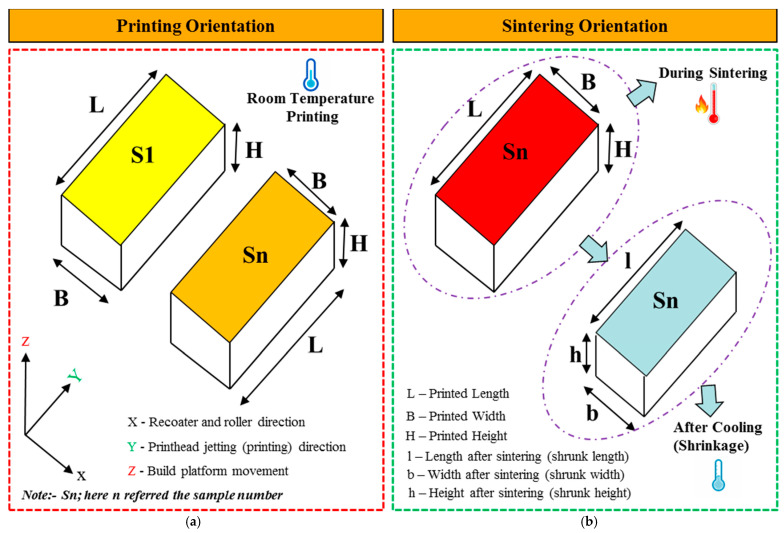
Illustrates the orientation of (**a**) printing and (**b**) sintering of the test coupons, along with the representation of shrinkage post-sintering followed by cooling.

In the experimental arrangement, a water-based binder named Aquafuse (BA005) was utilized as the binding agent to fuse adjacent layers on the ExOne Innovent binder jet system (ExOne, North Huntingdon, PA, USA). Additionally, a specialized cleaner provided by ExOne, identified as CLOO1, was employed to effectively eliminate any surplus binder adhering to the machine’s print head. The size of the green part, produced using the BJ machine, was rectangular blocks of 1-inch × 0.5-inch × 0.5-inch (length × height × width), as shown in Figure 3. All test parts were manufactured throughout the printing process with a uniform layer thickness of 100 μm and an oscillation speed ranging from 2600 to 2750 revolutions per minute (rpm). The recoating speed is adjusted within 20 to 28 mm per second (mm/s). In comparison, the roller and transverse speeds are set between 200 and 300 rpm and 12 and 14 mm/s, respectively. The binder saturation was set at 65%, with a binder set time of 10 sec and a drying time of 30 s. In this study, 18 coupons were produced, and the respective sample designation is mentioned in Table 2.

The green parts produced on the BJ underwent curing at 200 °C for 14 h in a Yamato drying oven (model DX402C, Yamato Scientific, Tokyo, Japan) with a ramp rate of 30 °C per minute, followed by the debinding and sintering process. The debinding and sintering processes were combined and executed simultaneously in a single step in a tube furnace (model TF1-1600, Carbolite Gero, Hope Valley, UK). The samples underwent debinding at 480 °C for 60 min and were then gradually heated to the designated sintering temperature. The sintering process was carried out at a constant heating rate of 15 °C per minute within a tube furnace filled with argon gas after evacuating the tube to a vacuum level of 1 × 10^−2^ millibar. Subsequently, the tube was pressurized with argon gas at a precisely controlled pressure of 12–15 Kilo Pascal (KPa).

The evacuation and argon-filling procedures were repeated at least five times before initiating the debinding and sintering steps. The specific parameters related to the curing, debinding, and sintering of all the samples are detailed in Table 2. Determining the appropriate sintering temperature for AISI M2 tool steel in a BJ process involves several key considerations that ensure optimal material properties, part geometry, and microstructure. The fundamental principle guiding this selection is achieving a suitable balance between densification and structural integrity and ensuring minimal shape distortion. The pseudo phase diagram for AISI M2 tool steel helps determine optimal sintering temperatures by indicating critical phase transitions [53,54]. At around 1300 °C, a combination of liquid (L), alpha-ferrite (α), and gamma-austenite (γ) is formed, promoting densification through capillary action. However, sintering at temperatures above 1300 °C can lead to excessive liquid, causing significant shape distortion due to increased fluidity [53]. Temperatures near 1280 °C offer a balanced phase composition with L, α, γ, and carbides (MxC), achieving adequate densification while retaining hardness and wear resistance from the carbides. At 1270 °C, the phase structure primarily consists of L, γ, and carbides (M_x_C), providing sufficient densification without excessive distortion risk [53]. This balance of phases at these temperatures is key to obtaining high-quality sintered parts with good mechanical properties.

After the sintering process, the samples underwent measurements to determine the shrinkage in each direction, as depicted in Figure 3. The length, height, and width were measured using a digital vernier caliper with a resolution of 0.01 mm before the samples were loaded into the tube furnace and after sintering and cooling. Ten measurements were obtained for each dimension (length, height, and width), and the average dimensions were used for calculation. The shrinkage of the test coupon was calculated using the following formula:

(1) $$Shrinkage\;\left(\%\right)=\frac{Printed\;Dimensions\;\left(L,B,\;H\right)-Dimension\;after\;sintering\;followed\;by\;cooling\;\left(l,b,h\right)}{Printed\;Dimensions\;\left(L,B,\;H\right)}\times 100$$

where L, B, and H are the dimensions of the test coupons before placing them in the tube furnace, and l, b, and h are the dimensions of the sintered samples after cooling, as shown in Figure 3. Subsequently, the specimens were cut using a low-speed precision saw (Allied High Tech, model TechCut 4XTM, Dominguez, CA, USA) and mounted using graphite-based conductive mounting powder procured from Allied High Tech Products, Inc., headquartered in Los Angeles, CA, USA. To attain a smooth surface finish, the specimens underwent polishing utilizing silicon carbide sheets (brand: BUEHLER, situated in Lake Bluff, IL, USA) with incrementally finer grit sizes, ranging from #120 to #1200 grid SiC sandpaper.

### 2.3. Scanning Electron Microscopy (SEM)

The specimens underwent polishing using a 0.04 µm colloidal silica suspension on a micro cloth to achieve the desired metallographic finish suitable for SEM characterization. Following this, the samples were cleaned with water, and then an ultrasonic cleaner (model 3800, Branson Ultrasonics, Danbury, CT, USA) was utilized to eliminate the silica residue from the surface. SEM (Inspect F50 system, FEI Corporation, Hillsboro, OR, USA) was employed to investigate the microstructural properties of the printed components. This analysis aimed to identify various carbides, assess the networked carbides’ shape and size, and analyze the specimens’ porosity. 

### 2.4. Digital Image Analysis

To quantitatively assess the connectivity of carbide networks within the metal matrix of M2 tool steel structures, a series of four scanning electron microscope (SEM) images were captured from random locations within each sample. These images were subsequently subjected to image processing methodologies tailored to discern and segment distinct regions indicative of carbides exhibiting varying colors, specifically M_6_C carbides (white), MC carbides (light grey), and porosities (black). This discernible feature of the microstructure facilitated the detection and quantification process. The acquired images were initially preprocessed through conversion to grayscale, a fundamental step in image analysis workflows. Grayscale conversion effectively reduced the images to a single channel, representing variations in intensity across the microstructure. Subsequently, the Python code implemented a thresholding technique to partition the grayscale image into discrete shades of grey, each corresponding to a specific color within the original image. The methodical framework employed a systematic approach encompassing grayscale conversion, thresholding, segmentation, quantification, and visualization to comprehensively analyze and quantify the spatial distribution of different color regions within the images. The code effectively distinguished between carbide phases and background features by assigning intensity thresholds, facilitating segmentation into distinct color regions indicative of carbide networks and other microstructural components. 

## 3. Binding Mechanism

**Figure 4:**
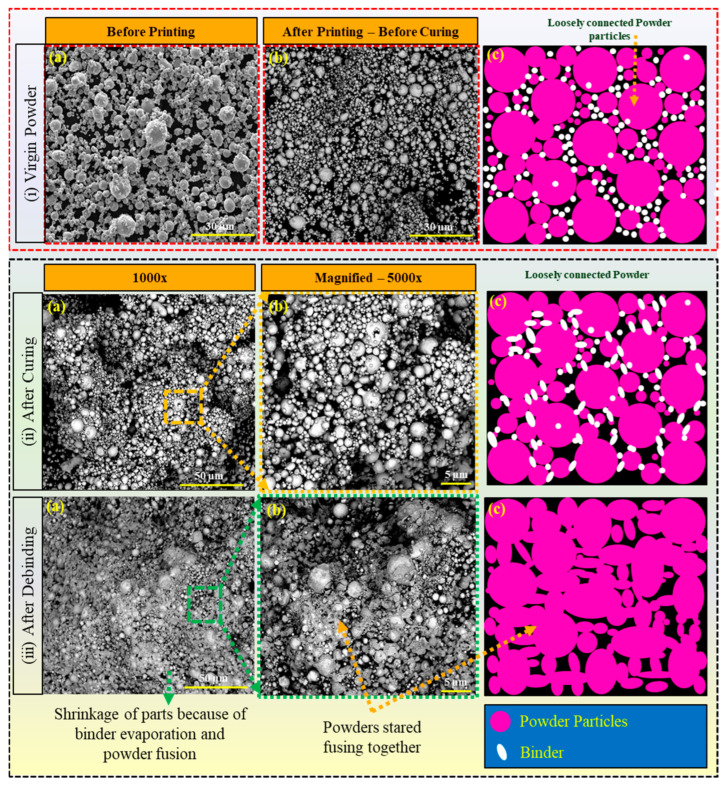
Illustration of binder–powder interaction throughout the different stages of binder jetting. (**i**) Printing at atmospheric temperature–the interaction between the binder and powder particles at room temperature during the initial deposition stage, (**ii**) after curing at 200 °C for 12 h, and (**iii**) after debinding at 480 °C for an hour.

The BJAM process entails the deposition of a liquid binding agent, also known as a binder, onto successive layers of powder material to construct a three-dimensional object [55]. The binding mechanism in binder jetting relies on the interaction between the liquid binder and the powder particles, governed by principles of wetting, capillary action, and adhesion [41,56,57]. At the onset of the printing process, the liquid binder is dispensed onto the powder bed through inkjet nozzles, precisely depositing droplets onto targeted regions based on the digital design. The binding agents employed in BJ processes intricately interlock within the porosity regions among the powder particles, as depicted in Figure 4i(b,c). Within this microenvironment, some binding agents achieve complete contact with the powder particles, facilitating strong adhesion and bonding, while others remain in a liquid state with minimal or no contact with the powder particles [58,59]. This dynamic interplay between the binding agents and powder particles plays a critical role in establishing the printed parts’ structural integrity and mechanical properties.

Upon contact with the powder particles, the binder undergoes a series of interactions facilitated by its physicochemical properties, such as surface tension. Wetting is the initial phenomenon where the binder spreads over the surface of the powder particles, driven by the reduction in surface energy [14,19]. The interfacial tensions between the binder, powder, and surrounding atmosphere influence this process. Capillary action further aids in the penetration of the binder into the interstitial spaces between adjacent powder particles, promoting cohesion within the printed layer. Ideally, the binder should exhibit good wettability towards the powder particles. This is often achieved by incorporating surface modifiers in the binder formulation [60]. These modifiers, typically surfactants or dispersants, can lower the binder’s surface tension, allowing it to spread and intimately contact the powder surface. Additionally, the binder can be formulated to exploit specific surface functionalities on the powder particles. During the curing process, as shown in Figure 4ii, when the green part is heated at a specific temperature (in this study, ~200 °C), the binder transforms from a liquid to a solid state with a few percentages of evaporation. In this process, the molecules of the binder infiltrate the powder, form larger, interconnected structures with the powder, and hold it tightly, as shown in Figure 4ii(c). As the temperature rises, the molecules in the binder gain energy, which promotes chemical reactions that form these larger structures. As the binder infiltrates the powder bed, the adhesion mechanism comes into play, establishing bonds between the binder and powder surfaces [61,62]. Van der Waals forces, electrostatic interactions, and chemical bonding contribute to the adhesive forces between the binder and powder particles [63]. The specific nature of these interactions depends on the binder formulation’s properties and the powder material’s surface characteristics [64]. During the debinding process at 480 °C, the binder undergoes a phase change from a solid to a gaseous state through a process known as evaporation or sublimation (from liquid to gas if the binder is not solidified during curing) [65]. This occurs because the temperature exceeds the boiling or sublimation point of the binder material, causing it to transition directly into vapor form. As the binder evaporates, it escapes from the porous structure formed by the powder particles, leaving behind voids previously occupied by the binder. Simultaneously, the powder particles begin to fuse as a result of the elevated temperature, as shown in Figure 4iii. At 480 °C, the powder particles may undergo sintering, where adjacent particles come into contact and form necks between them. These necks gradually grow as the particles continue to be heated, promoting the consolidation of the powder particles. As sintering progresses, the gaps between particles diminish, and the overall density of the material increases. Densification, or the increase in material density with minimal or no binder, is a key outcome of the debinding process. As the binder evaporates and the powder particles fuse together, the void spaces within the part are reduced, leading to a more compact and solid structure and, hence, the shrinkage.

## 4. Defect Analysis in Binder Jetting AM

### 4.1. Importance of Defect Analysis for Quality Control

**Figure 5:**
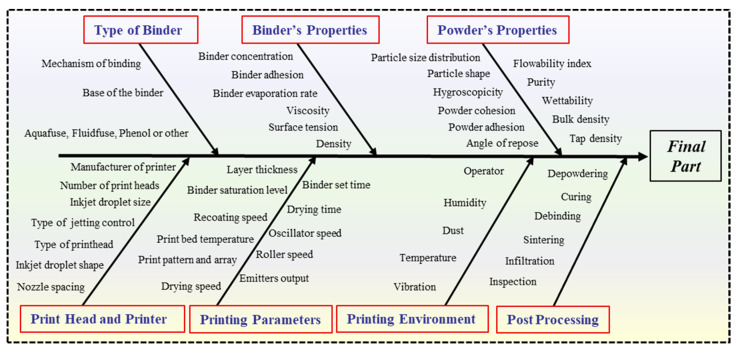
Fishbone (Ishikawa) diagram illustrating the critical factors that influence the quality of the final printed component in binder jetting.

Defect analysis is crucial for optimizing the manufacturing process, improving part quality, and ensuring consistency in production. Defect analysis provides valuable insights into the underlying mechanisms contributing to defects in the BJ process. By systematically analyzing the types and causes of defects, engineers and researchers can better understand the complex interactions between various process parameters, material and mechanical properties, and environmental conditions. Defects are inherent to the BJ process and can arise due to multiple factors related to material properties, process parameters, and machine performance throughout the manufacturing process [26]. These defects manifest in different forms, including porosity, warping, surface roughness, and incomplete bonding between powder layers [14,66]. Also, defects such as misalignment, layer shifting, and nozzle clogging can occur due to mechanical issues or inconsistencies in process parameters. Figure 5 illustrates a graphical representation displayed on a fishbone (or Ishikawa) diagram, highlighting the factors determining the ultimate quality of the printed components. Therefore, effective quality control measures and optimization of process parameters are essential for minimizing defects and ensuring the production of high-quality printed parts. One key aspect of defect analysis is identifying the root causes of defects. This involves investigating factors such as binder distribution, powder characteristics, curing parameters, printing environment, print head and printer type, and post-processing techniques. Additionally, defect analysis plays a crucial role in quality assurance and certification processes. This is particularly important in industries such as aerospace, automotive, and medical devices, where components’ structural integrity and functionality are critical [67,68]. Defect analysis is essential for quality control in BJ as it systematically identifies, understands, and mitigates factors that can affect part quality. In the field of AM, researchers continually develop innovative tools to ensure the quality of manufactured components. Li et al. [69] introduced AM-SegNet, a deep learning model designed to rapidly and accurately segment high-resolution synchrotron X-ray images in metal AM. This model achieved an impressive 96% accuracy, with processing times below four milliseconds per frame, allowing for efficient identification and analysis of critical features like keyholes and pores, thereby advancing our understanding of AM processes. Campbell et al. [70] developed a visualization tool to analyze the surface roughness of these components through image analysis. Similarly, Tapia et al. [71] devised a model to predict porosity on selective laser melting (SLM) surfaces using spatial Gaussian processes. This sophisticated mathematical technique leverages image analysis to improve predictive accuracy. Syam et al. [72] introduced an image-monitoring system capable of detecting surface quality and tracking AM parts during manufacturing. While these techniques are highly effective for laser powder bed fusion (LPBF), where sintering occurs during printing, they are less applicable to BJ, where sintering occurs in a separate furnace, complicating the detection of carbide formation. So, the best way to achieve this is to minimize the defects in the printed part (green part). Despite these limitations, BJ-produced sintered parts possess quasi-isotropic properties, allowing researchers to correlate carbide structures across multiple layers to the entire component’s volume. Researchers sectioned the sintered parts to overcome the challenges of studying these carbides in BJ-produced parts and captured SEM images at various locations. These carbides were then segmented into four distinct colors to facilitate analysis and categorization, providing a detailed spatial distribution of microstructural characteristics. This innovative approach allows for a deeper understanding of the sintered parts’ microstructural properties, offering valuable insights for further process optimization.

### 4.2. Classification of Common Defects in Binder Jetting AM Parts

**Figure 6:**
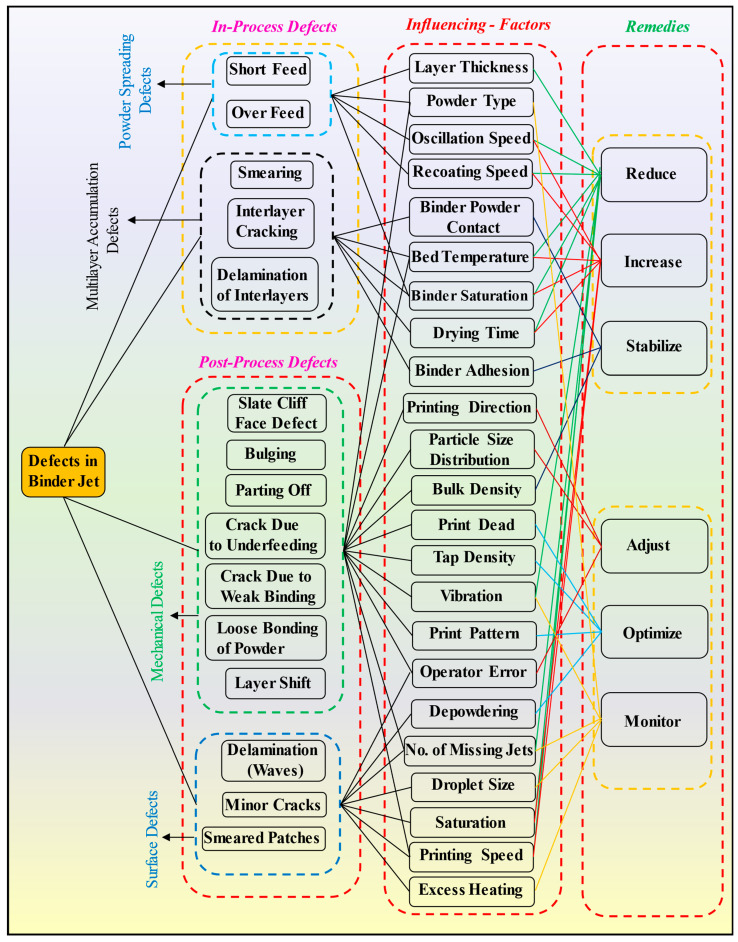
Classification of the common defects and their relationship with the influencing factors and remedies.

BJAM, along with other powder-bed-based (PBB) techniques such as laser powder bed fusion (LPBF) and selective laser sintering (SLS), offers immense potential for fabricating complex parts with high geometric accuracy [30,73,74,75,76]. However, attaining defect-free, dense, uniform layers remains a paramount challenge. Particularly in BJ, where the green parts exhibit low strength during printing, even minor defects can compromise the integrity of the final product. Figure 6 illustrates the categorization of typical defects, their correlation with the factors influencing them, and the corresponding solutions to address these issues. 

In this investigation, the defects observed throughout the printing process of test specimens via the BJ are categorized primarily into two distinct segments. Initially, defects discernible during the printing procedure are designated as in-process defects, while those identified after printing and curing procedures are denoted as post-process defects. In the realm of in-process defects, the problems are either caused by powder spreading or multilayer accumulation of various defects, such as smearing. On the other hand, post-process defects, which are usually identified visually, include both mechanical and surface defects. Parts with mechanical defects are simply thrown away and cannot be used further. However, parts with surface problems like minor cracks, delamination, and occasional smearing spots may still be suitable for sintering, depending on how significant the problems are.

### 4.3. In-Process Defects and Remedies

In-process defects arise during the actual printing operation. One common type of in-process defect is related to powder spreading, where inconsistencies or irregularities in powder deposition lead to defects such as insufficient powder distribution or non-uniform layer thickness. The following are the defects that may arise due to improper powder spreading.

#### 4.3.1. Short- and Over-Feeding

**Figure 7:**
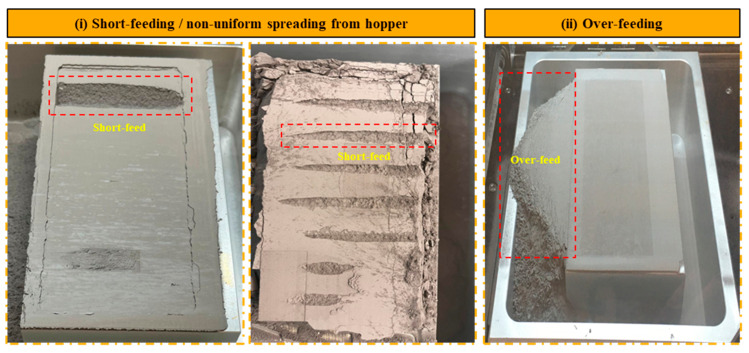
Illustration of defects arising from (**i**) short-feeding or non-uniform powder discharge from the hopper and (**ii**) over-feeding of the powder from the hopper.

The flowability of the powder material itself can significantly impact the occurrence of short-feeding defects [77]. Factors such as particle shape, surface roughness, and electrostatic charge can influence the flow behavior of powder particles within the printing system [78,79]. Poor powder flowability can impede the smooth movement of powder material through the hopper and onto the build platform, resulting in inadequate powder deposition and short-feeding defects in the printed parts. Figure 7i illustrates short-feeding defects in binder jet printing, primarily attributed to insufficient distribution of powder particles across the build area. This defect arises from several factors, including suboptimal oscillation speed and insufficient powder discharge from the hopper. Additionally, powder clogging at specific sections of the labyrinth plate can impede the smooth flow of powder, exacerbating the issue. Furthermore, when the recoating speed of the roller is too high, there is no sufficient time for powder spreading, resulting in improper distribution in the build zone. Another influential factor observed was the particle size distribution of the powder material used in the printing process. Variations in particle size distribution can influence the flow behavior of the powder, leading to irregular spreading patterns and inadequate powder deposition in specific areas of the build platform. Various studies have suggested that powders with smaller particle sizes (<20 microns) may experience difficulties in powder flow [80,81,82,83], exacerbating short-feeding defects and compromising part quality [84,85,86]. Over-feeding, depicted in Figure 7ii, commonly occurs when there is excessive powder discharge from the hopper during the binder jet printing process. This issue is particularly problematic for larger-sized parts, as the surplus powder can lead to various adverse effects on print quality.

To mitigate the short-feeding defect effectively, it is essential to carefully select powder with an appropriate size distribution, typically ranging from 20 to 60 microns. The other best remedy is to increase the oscillation speed, which will increase the powder discharge from the hopper. To address the issue of short-feeding, the oscillation speed was increased to 2750 rpm, and the recoating speed was lowered to 20 mm/s, leading to a greater volume of powder discharged from the hopper. Conversely, the oscillation speed was reduced to 2600 rpm to correct over-feeding. Certain powders, such as AISI M2 tool steel, exhibit high sensitivity to moisture content, which can exacerbate short-feeding issues. In this study, efforts were made to enhance the powder flowability index by employing two main techniques: baking and vacuum treatment. Baking the powder at approximately 150 °C for 2 to 3 h proved to be another effective method for reducing moisture content without inducing oxidation. This controlled baking process helps remove excess moisture from the powder, improving its flowability and reducing the likelihood of short-feeding defects. Notably, the baking temperature is carefully maintained below the curing temperature to prevent any undesirable oxidation of the powder material.

Alternatively, placing the powder in a vacuum environment with a pressure below ten bar absolute for approximately one hour can also help to reduce moisture content. Vacuum treatment facilitates moisture removal by creating a low-pressure environment, allowing trapped moisture within the powder particles to escape. This method offers a non-oxidative approach to remove moisture, thereby preserving the integrity of the powder material. By implementing these remedies, manufacturers can effectively address short-feeding defects in binder jet printing processes, ensuring smoother powder flow and improved print quality.

#### 4.3.2. Multilayer Accumulation Defects

**Figure 8:**
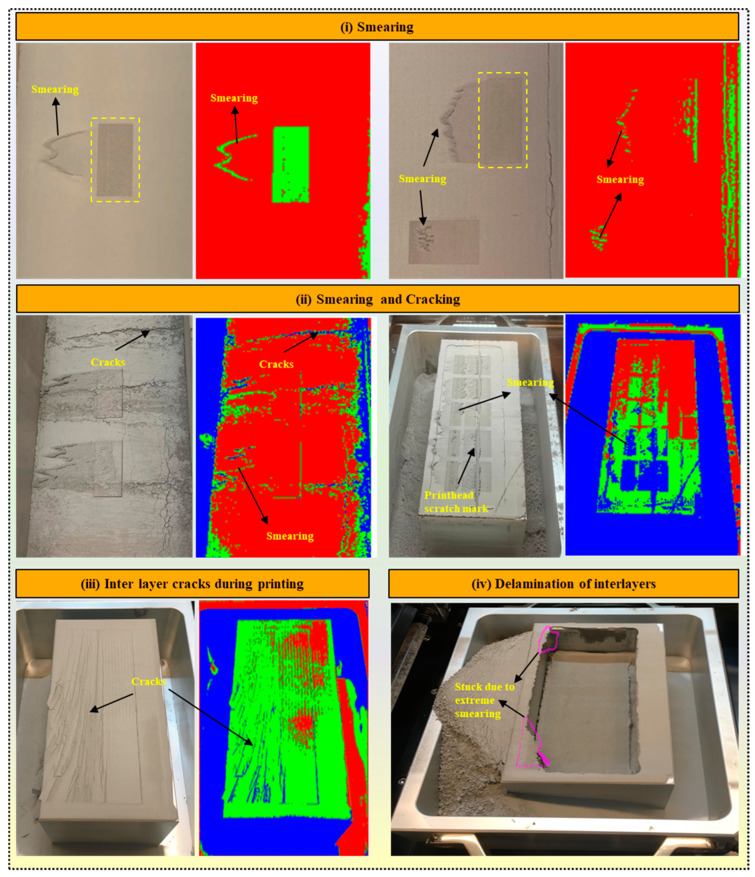
Illustration of multilayer accumulation of defects, (**i**) smearing, (**ii**) smearing along with the cracks in the build zones, (**iii**) interlayer cracks in the print zone, and (**iv**) delamination of interlayers.

Once an optimal print pattern is achieved through binder deposition, it is essential to recognize that perfection in a single layer does not guarantee a flawless final printed product. The accumulation of defects layer by layer can introduce unpredictability during printing. In binder jet printing, the gradual buildup of defects between layers often results from inaccurately chosen process parameters. Researchers have dedicated significant efforts to address and mitigate these issues [87,88,89]. In this study, various types of multilayer defects observed are illustrated in Figure 8. Each defect is explained below, along with possible methods to tackle them:

1. Smearing: In BJ, smearing occurs when the freshly recoated powder layer comes into contact with the partially dried binder of the previous layer during the printing process when the roller levels the powder in the build zone. This contact can lead to the unintentional spreading or smudging of the binder across the surface of the powder bed, resulting in irregularities or defects in the printed part, as shown in Figure 8i. Therefore, the binder’s proper drying temperature and duration play an essential role in smearing. Smearing during printing often occurs due to several other factors [90]. Firstly, it is observed that excess binder application leads to smudging when the recoated powder layer is deposited. If the binder is applied excessively or in large droplets, it can spread beyond the intended boundaries, compromising the integrity of the printed layers. Secondly, binder saturation contributed to smearing. When the binder saturates the powder particles excessively, the freshly applied binder may encounter previously saturated particles, leading to easier spreading and smearing along the powder bed surface. Furthermore, it is also observed that suboptimal powder spreading can result in uneven distribution of the binder. If the powder spreading mechanism fails to distribute the powder evenly or if irregularities exist in the powder bed surface, the binder may not be uniformly absorbed. This uneven distribution can cause localized areas of excess binder, increasing the risk of smearing when the next layer is applied.

Optimizing the drying time, drying temperature, and saturation percentage of the binder is essential to effectively reduce or eliminate the smearing defect. Proper drying allows the powder to absorb the binder adequately and ensures it is sufficiently dry for subsequent layers to be applied. This study’s first step towards mitigating smearing involved optimizing the binder saturation level for printing, particularly for the initial ten layers. Ensuring that the binder saturation was optimal (65% in this study), the drying time was 30 s, and the bed temperature was 60 °C reduced the likelihood of smearing during printing significantly. To further prevent smearing in consecutive layers, the temperature of the print bed was regulated to approximately 50–60 °C, and a specific time duration of 15–20 s was implemented (this value may vary from material to material). These controlled conditions facilitated the drying of the binder between layers, minimizing the risk of smudging and improving the overall print quality.

2. Smearing and cracking: Smearing and cracking defects typically occur when the powder bed density is insufficient, leading to inadequate adhesion between powder particles and ineffective binder penetration. We observed that when the powder bed density was not optimal, there were gaps and voids between the powder particles, resulting in poor inter-particle bonding, as shown in Figure 8ii. As a result, the binder applied during printing may not effectively penetrate and bind the powder particles, leading to incomplete consolidation of the printed layers. This inadequate bonding can result in the smearing of the printed features and may also contribute to cracking as the part undergoes post-processing or handling. Another contributing factor to smearing and cracking is the excessive adhesion of the binder to the roller mechanism during printing [91]. If the roller becomes coated with an excess amount of binder, it may unevenly transfer this excess binder onto the powder bed, leading to localized areas of high binder concentration. Consequently, these areas may exhibit excessive consolidation and poor powder adhesion, resulting in smearing of the printed layers and potential cracking upon curing or post-processing. To mitigate this defect, the best remedies are increasing the oscillation speed, reducing recoating and roller speed, and increasing the temperature of the build zone and drying time. In this study, the optimized parameters to mitigate smearing and cracking issues included an oscillation speed of 2600 rpm, a recoating speed of 20 mm/s, a roller speed of 200 rpm, a build zone temperature of 60 °C, and a drying time of 30 s.
3. Interlayer cracks during printing: Interlayer cracks in binder jet printing were observed when regions of low powder density failed to adequately support the deposition of subsequent layers, leading to cracks between layers, as shown in Figure 8iii. This is primarily caused by inadequate powder compaction and uneven binder distribution within the powder layers. Factors contributing to interlayer cracks included improper powder spreading, inconsistent binder application, and insufficient drying or curing of the binder. Addressing interlayer cracking requires optimizing various process parameters, such as uniform powder spreading, precise binder application, and adequate drying or curing times between layers. Additionally, controlling printing parameters like bed temperature and build plate movement speed can minimize and reduce the likelihood of interlayer cracks. By carefully managing these factors, the quality and integrity of printed parts can be improved, resulting in fewer interlayer defects and enhanced overall print quality. In this study, to address deficiencies and ensure uniform powder spreading, the recoating speed was reduced to 22 mm/s with a roller speed of 250 rpm, a binder set time of 10 s, and a drying time of 30 s. To ensure precise binder application, a missing jet test was performed every 4 h during printing, but this was only applied for larger blocks with a volume exceeding 1500 mm^3^.

4. **Delamination of the interlayer**: It occurred when certain portions of the print adhered to the build plate during the downward movement of the plate, as shown in Figure 8iv. This phenomenon is typically observed in binder jet printing. It is attributed to smearing at the boundaries of the build zone, which causes adhesion between the printed layers and the build plate surface. When the build plate descends to accommodate the deposition of subsequent layers, regions of the print with plate corner adhesion fail to detach from the build plate, resulting in delamination. Several factors contribute to interlayer delamination in binder jet printing, such as excessive smearing, insufficient drying, very low transverse speed, and high roller speed when the hopper assembly returns to its home position. To tackle this problem, the best remedy is to control the printing parameters, especially the binder set time (increase), drying time (increase), roller speed (decrease), and recoating speed (decrease). The optimized experimental parameters include a binder set time of 15 s, a drying time of 40 s, a roller speed of 200 rpm, and a recoating speed of 20 mm/s.

### 4.4. Post-Process Defects

Post-process defect identification is a crucial step in BJ to enhance the quality control of the process for several reasons. Firstly, while in-process monitoring and control can help detect and mitigate defects during printing, not all defects may be immediately apparent until after the printing and post-processing steps are completed. Therefore, a thorough examination of the printed parts after post-processing allows for comprehensive defect identification and analysis. Secondly, post-process defect identification provides valuable feedback for process optimization and improvement. Users can pinpoint areas of weakness in their BJ process by identifying and documenting the types and frequencies of defects encountered in printed parts. This information enables them to refine their printing parameters, adjust material formulations, optimize post-processing techniques, and implement corrective actions to reduce the occurrence of defects in future prints. Defects like cracks, delamination, and dimensional inaccuracies can impact mechanical properties and surface finish [23]. Identifying these defects post-processing allows corrective measures such as heat treatment or surface finishing to meet quality standards. Additionally, it ensures the integrity and functionality of parts for critical industries like aerospace and medical, where defects could pose potential safety risks [92]. Overall, it enables comprehensive defect analysis, process optimization, and validation of printed parts for their intended applications. Below are the explanations of the various types of post-process defects observed in this study.

#### 4.4.1. Mechanical Defects

Mechanical defects in binder jetted parts refer to structural flaws or irregularities that compromise the mechanical integrity or performance of the printed components. These defects can manifest in various forms, including cracks, porosity, warping, and dimensional inaccuracies. They often arise due to inadequate powder consolidation, improper binder distribution, insufficient curing, and suboptimal process parameters. Addressing mechanical defects is crucial to ensure binder jetted parts’ reliability, durability, and functionality in various applications. These defects are typically identified through visual inspection and further evaluation. Mechanical defects, including slate cliff faces, bulging, cracks, weak binding, and layer shifts, are among the primary concerns in this category. Parts exhibiting significant mechanical defects are usually deemed unsuitable for further use or additional processing.

**Figure 9:**
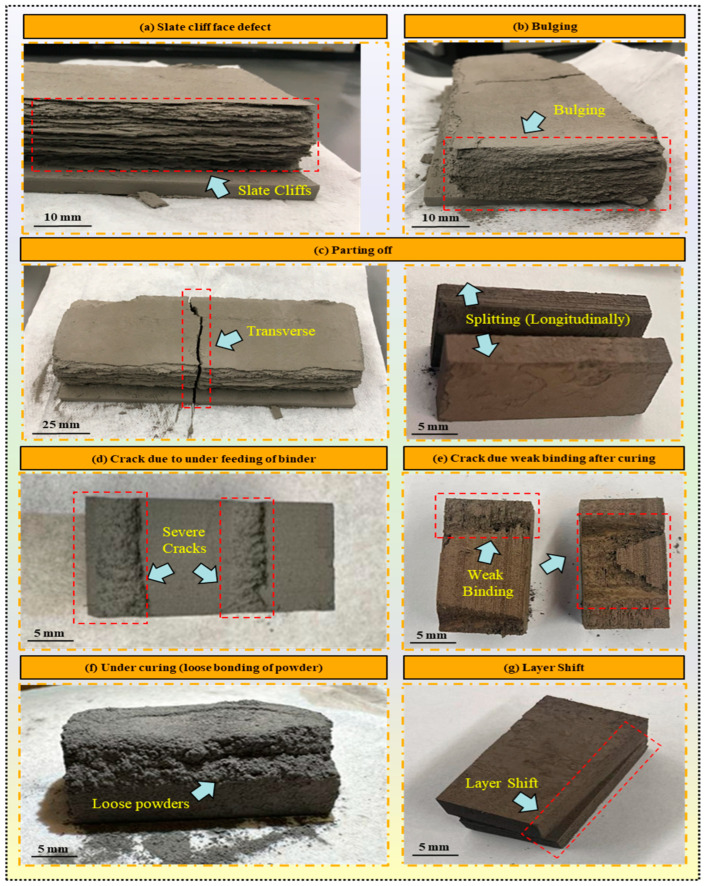
Illustration of mechanical defects observed post curing: (**a**) slate cliff face defect, (**b**) bulging, (**c**) parting off, (**d**) crack due to under-feeding, (**e**) crack due to weak binding, (**f**) loose bonding of the powder particles, and (**g**) layer shift.

1. Slate cliff face defect: The “slate cliff face” defect observed in binder jet printing refers to a distinctive surface irregularity resembling the rugged face of a slate cliff, as shown in Figure 9a. This defect manifests as uneven and jagged layers or edges along the printed part’s surface, resembling the natural fissures and contours of slate rock formations. The irregularities can occur due to various factors during the printing process, including insufficient powder compaction, excessive smearing between the layers, and inconsistent binder application due to inadequate adhesion between layers. These inconsistencies can compromise the printed part’s structural integrity and aesthetic appearance, necessitating corrective measures to mitigate the defect. The parts with these kinds of defects cannot be processed for debinding and sintering. To address the slate cliff face defect, optimization of process parameters such as powder spreading, binder saturation, and drying time conditions is essential. Ensuring uniform powder compaction and consistent binder penetration across the build area can help minimize surface irregularities and enhance the overall quality of the printed part. To address this defect, the optimized parameters include an oscillation speed of 2650 rpm, a binder saturation of 65%, a drying time of 45 s, and a binder set time of 5 s. Additionally, to ensure proper compaction of the powder, the recoating speed was reduced to 20 mm/s.
2. Bulging defect: The “bulging” defect in binder jetting refers to a characteristic irregularity where certain sections of the printed part exhibit pronounced outward protrusions or swelling, resembling bulges or deformities, as shown in Figure 9b. This defect typically occurs due to localized variations in powder compaction and binder saturation during printing, leading to non-uniform material deposition and layer buildup. Other factors include inadequate powder spreading and leveling mechanisms, uneven binder distribution, or insufficient adhesion between adjacent layers. In areas where the powder bed is not uniformly compacted or where excessive binder is deposited, the excess material can accumulate and cause the formation of bulges or raised regions on the part’s surface. Optimizing recoating speed and compaction mechanisms (roller speed) to mitigate the bulging defect is essential to ensure uniform material deposition across the build area. Fine-tuning binder application parameters and controlling the curing process can help minimize excess material buildup and promote better adhesion between layers. Implementing quality control measures and conducting thorough inspections during and after the printing process can aid in identifying and addressing bulging defects promptly, thus improving the quality and dimensional accuracy of binder jetted parts. The most effective approach to minimize bulging is to discharge excess powder from the hopper. The optimized parameters for this include an oscillation speed of 2750 rpm, a recoating speed of 20 mm/s, and a roller speed of 200 rpm, ensuring uniform spreading and optimal compaction of the powder in each layer.
3. Parting off: The “parting off” defect in BJ refers to the unintended separation of the printed part along its transverse (Figure 9c) or longitudinal (Figure 9d) direction, resulting in the formation of distinct sections or pieces. This defect typically occurs due to inadequate strength or bonding between adjacent layers, leading to structural instability and eventual detachment in the transverse direction, as shown in Figure 9c. Parting off may occur when the printed part lacks sufficient strength to withstand external forces or stresses applied perpendicular to its build layers, either due to its own weight or as a result of user error during depowdering. Weak interlayer bonding or insufficient material density in some areas of the part can contribute to this defect, causing the part to separate along its cross-section. Conversely, parting off in the longitudinal direction may occur when there are missed or inadequately bonded layers within the part’s structure. If specific layers fail to adhere correctly during the printing process or if there are inconsistencies in binder application, these weak points can become prone to separation over time, especially during depowdering; the parts become splatted longitudinally due to their own weight. Optimizing BJ parameters is essential to mitigate parting off defects in binder jetting, along with carefully monitoring printing layers susceptible to higher levels of smearing. When observing increased smearing in specific layers, it is advisable to extend the drying duration to dry the binder effectively. In this study, to counteract smearing issues, the drying time for problematic layers was increased by about 50–60% compared to standard layers, extending to 45–50 s. Additionally, the binder set time was shortened to 5 s to further reduce the risk of smearing and ensure more consistent layer quality.

4. Cracks due to under-feeding of binder and weak binding: Cracks stemming from under-feeding and weak binding in BJ are often a result of inadequate binder penetration and poor bonding between powder particles. Under-feeding occurs when the binder application fails to supply enough binder to thoroughly saturate the powder bed, resulting in inadequate adhesion between adjacent layers [93]. This study noted that the left half of the printhead jets became blocked during the last few layers, possibly due to contact with smeared surfaces, thus preventing the binder from being ejected onto those specific sections, as depicted in Figure 9d. On the other hand, weak binding arises when the binder-to-powder ratio is too low or when the binder fails to sufficiently penetrate the powder layers, resulting in weak bonds between layers, as shown in Figure 9e. These factors collectively contribute to cracks in binder jetted parts and underscore the importance of optimizing BJ parameters and post-processing procedures to mitigate such defects and enhance part quality. Therefore, the most effective remedies to address these defects involve monitoring the off jets of the print head nozzles, a task easily performed during printing. If specific sections of the part are not adequately wetted with the binder, it may indicate issues with the off jets of the nozzles. The oscillation speed was raised to 2750 rpm to mitigate under-feeding issues while reducing the recoating speed to 20 mm/s. When lines were observed in the printed layers, a missing jet test was conducted to maintain uniform binder distribution. By promptly identifying and addressing these nozzle-related issues, such as cleaning or adjusting the nozzles as needed, the operator can ensure consistent and uniform binder deposition, thereby mitigating the risk of under-feeding and weak binding defects in binder jetted parts.
5. Under-curing: Under-curing in BJ refers to the insufficient curing or solidification of the binder material used to bind the powder particles together during the printing process. This defect occurs when the curing conditions, such as temperature or time, are not optimized, or the curing process cannot proceed for a sufficient duration. In this study, when insufficient curing duration of approximately 8 h was applied, it resulted in inadequate bonding of the powder particles by the binder material. Consequently, the printed parts exhibited a clumpy appearance, with the powder particles not fully bonded together, as shown in Figure 9f. Without sufficient bonding between the powder particles, the loosely bound powder may be susceptible to erosion or detachment from the part’s surfaces, especially during handling or subsequent processing steps. To address under-curing in BJ, it is essential to ensure that the curing process is carried out for a suitable duration and under optimized conditions. This may involve adjusting parameters such as curing temperature (180–220 °C) and time (12–16 h) to achieve complete and uniform solidification of the binder throughout the printed part. In this study, the curing temperature was set between 180 °C and 220 °C, with curing times ranging from 12 to 16 h, ensuring complete and uniform solidification of the binder throughout the printed part.
6. Layer Shift: Layer shift in binder jetting occurs when successive layers of the printed part are misaligned or displaced from their intended positions [24,41], leading to inaccuracies in the final part geometry, as shown in Figure 9f. In the presented case, the observed layer shift is attributed to several factors related to the drying process. The initial layers at the bottom of the printed part experienced shifting due to excess heat during drying. High temperatures can accelerate the drying process, causing the binder to solidify too quickly and resulting in an uneven distribution of material. Consequently, the roller, responsible for spreading the powder evenly, may push the layer away from the intended position, leading to lateral displacement or shifting. To address layer shifts in BJ, optimizing the drying parameters (drying temperature and time) to achieve uniform material distribution and minimize the risk of overheating is essential. Adjustments to temperature and drying duration can help mitigate the effects of excess heat and ensure consistent layer deposition throughout the printing process. Additionally, monitoring the printing environment and implementing quality control measures can help identify and address issues such as layer shift in a timely manner, ensuring the production of high-quality printed parts. To address the issue of layer shift, the bed temperature was set at 50 °C with a drying time of 25 s.

#### 4.4.2. Surface Defects

Although they are less severe than mechanical defects, surface defects can still impact the part’s aesthetics and functionality. These may include minor cracking, delamination, or irregular patches of smearing on the surface. 

**Figure 10:**
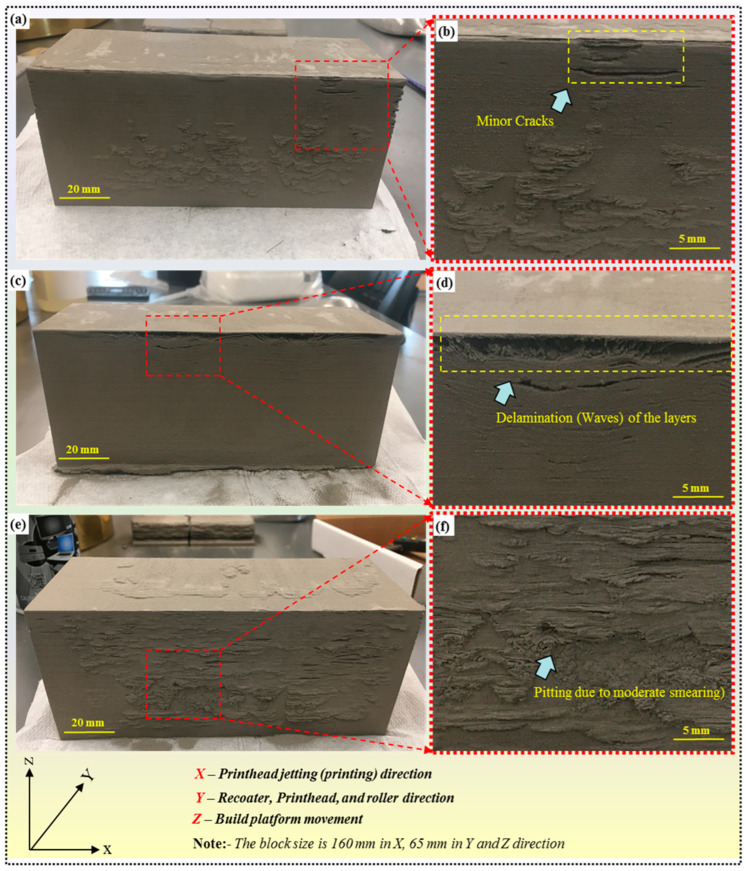
Illustration of the surface defects observed in the brown parts (after curing), (**a**,**b**) parts with minor cracks, (**c**,**d**) parts with spotted delaminated layers, and (**e**,**f**) parts with sporadic patches of smearing.

Despite these imperfections, parts with surface defects may still be rectifiable depending on the specific application and tolerance requirements. Surface defects in binder jetted parts refer to imperfections or irregularities on the outer surface of the printed part. Although not as critical as mechanical defects, surface defects can affect the part’s appearance, functionality, and mechanical properties. Minor cracking appears as small fractures or fissures on the surface of the printed part, often caused by inadequate bonding between layers or excess powder removal during depowdering, as shown in Figure 10a,b. As illustrated in Figure 10c,d, delamination occurs when layers of material separate from each other, resulting in a layered or peeling effect on the surface. Sporadic patches of smearing refer to localized areas where excess binder has spread unevenly, as shown in Figure 10e,f, leaving irregular deposits or streaks on the part’s surface. While surface defects may compromise the aesthetics of the printed part, their impact on functionality depends on the specific application and tolerance requirements. Minor surface defects may sometimes be acceptable, especially for non-critical applications or prototypes. However, for parts intended for functional use or visual appeal, surface defects may need to be addressed to ensure optimal performance and appearance.

To mitigate surface defects in BJ, the optimization of printing parameters such as binder application, powder spreading, and curing conditions is essential. Fine-tuning these parameters can help to achieve more uniform material deposition and bonding between layers, reducing the likelihood of surface imperfections. Additionally, post-processing techniques such as sanding, polishing, or surface texturing may be employed to improve the surface finish and mask minor defects, enhancing the overall quality of the sintered part.

### 4.5. Shrinkage in the Sintered Parts

**Figure 11:**
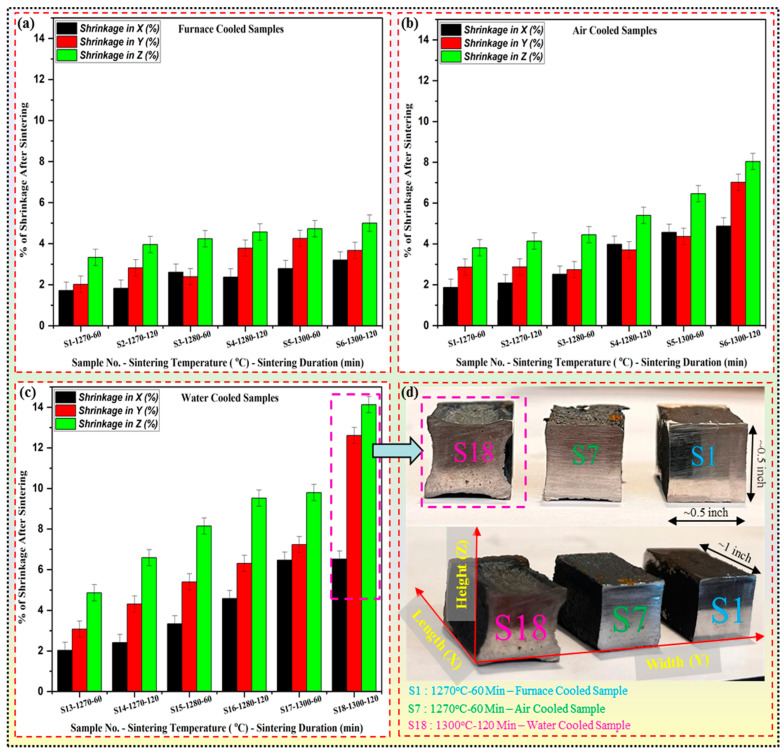
The percentage of shrinkage observed in the test coupons printed via binder jetting after sintering along the length (*X*), width (*Y*), and height (*Z*) axes for (**a**) furnace-cooled, (**b**) air-cooled, and (**c**) water-cooled samples. Additionally, (**d**) illustrates the appearance of samples after sintering to visually demonstrate the shrinkage.

During the sintering process of a rectangular block (0.5-inch × 0.5-inch × 1-inch) fabricated via BJ, shrinkage occurs due to various underlying mechanisms. Initially, as the part undergoes heating, the binder material within the powder bed evaporates or decomposes, removing organic components and leaving behind voids or pores. This process contributes to a reduction in volume and mass within the part. Additionally, as the temperature rises, the powder particles within the block undergo plastic deformation and densification. This leads to a rearrangement of powder particles, causing them to pack more closely together. Consequently, the voids left by the evaporated binder are gradually filled, further reducing the overall volume of the part. Moreover, as sintering progresses, interparticle diffusion becomes prominent, allowing atoms to migrate across particle boundaries and facilitating the formation of necks between adjacent particles [94]. This necking phenomenon decreases pore size and increases particle coalescence, ultimately leading to densification and shrinkage of the part. The bar chart represented in Figure 11 shows the shrinkage percentages along the *X* (length), *Y* (width), and *Z* (height) axes for three different cooling conditions: furnace-cooled, air-cooled, and water-cooled. Each bar corresponds to the average shrinkage value obtained from multiple measurements for each cooling condition. It is interesting to note a gradual increase in shrinkage percentages across all axes from furnace-cooled to air-cooled to water-cooled conditions. This trend indicates that the cooling rate has a pronounced effect on the shrinkage behavior of the binder jetted parts during sintering. 

The average shrinkage percentages along the *Z* axis are consistently higher than those along the *X* and *Y* axes. For instance, as shown in Figure 11a, the average shrinkage along the *Z* axis ranges from approximately 3.3 ± 0.4% to 5.09 ± 0.4%, while the shrinkage along the *X* and *Y* axes ranges from approximately 1.7 ± 0.4% to 4.2 ± 0.4% in furnace-cooled samples. This trend can be attributed to the gravitational force acting vertically, contributing to more excellent compaction and densification along the *Z* axis, leading to increased shrinkage in this direction. Under air cooling conditions, similar trends in shrinkage behavior are observed, as shown in Figure 11b. The average shrinkage along the *Z* axis ranges from approximately 3.8 ± 0.4% to 8.06 ± 0.4%, whereas the shrinkage along the *X* and *Y* axes ranges from approximately 1.89 ± 0.4% to 4.9 ± 0.4%. In the case of water cooling, the shrinkage rate was very high, and as a result, a very high percentage of shrinkage was observed as illustrated in Figure 11c. The average shrinkage along the *Z* axis ranges from approximately 4.9 ± 0.4% to 14.1 ± 0.4%, whereas the *X* and *Y* axes range from approximately 2 ± 0.4% to 6.5 ± 0.4%.

In BJ, it is not feasible to completely eliminate shrinkage [95,96], but it can be effectively controlled and minimized through careful optimization of sintering parameters (sintering temperature and time) and the use of controlled environments (atmosphere using inert gases like nitrogen or argon or vacuum) with regulated cooling rates. Furthermore, employing controlled cooling rates during the sintering process is crucial for managing shrinkage. Gradual cooling can help alleviate thermal stresses and reduce the likelihood of distortion or cracking in the final part. By carefully managing these factors, users can achieve greater consistency and precision in their additive manufacturing processes, ultimately leading to higher-quality end products.

## 5. Carbide Quantification in Binder Jetting AM

### 5.1. Understanding Carbides in AISI M2 Tool Steel

**Figure 12:**
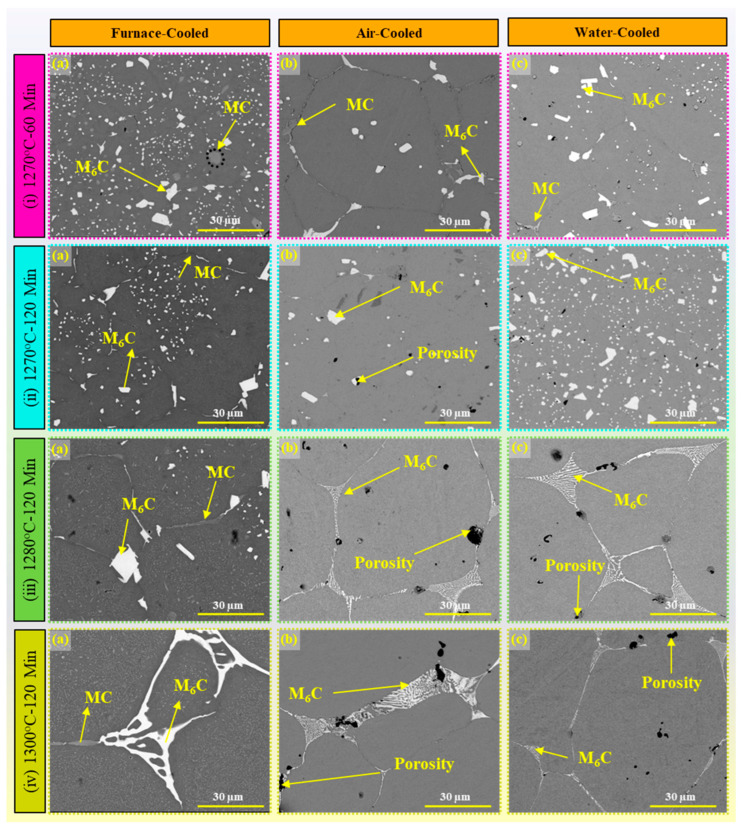
The microstructural images of AISI M2 tool steel processed on binder jetting (**i**) at 1270 °C for 60 min, (**ii**) at 1270 °C for 120 min, (**iii**) at 1280 °C for 120 min, and (**iv**) 1300 °C for 120 min followed by (**a**) furnace, (**b**) air and (**c**) water cooling.

Carbides play a crucial role in the microstructure of tool steel, imparting essential mechanical properties vital for its performance in various applications. Carbides act as strengthening agents within the steel matrix, providing resistance to deformation and wear during cutting, machining, and forming processes [97]. Understanding carbide composition, morphology, and distribution is essential for optimizing tool steel components’ microstructure and mechanical properties. Figure 12 shows the microstructure of AISI M2 tool steel processed on BJ. From this image, it can be clearly seen that at 1270 °C sintering temperature, most of the carbides are in the form of plates (Figure 12i,ii).

In contrast, in the case of higher sintering temperatures and duration (1280 and 1300 °C for 120 min), the carbides form a spider web kind of structure (Figure 12iii,iv). These carbides include MC, M_6_C, and M_2_C, each with distinct chemical compositions and morphologies. MC carbides, characterized by their light grey coloration, typically exhibit a distinctive fan-like structure within the microstructure of tool steel. These carbides are characterized by high concentrations of vanadium (V), tungsten (W), and molybdenum (Mo), while M_6_C carbides appear white in color and exhibit various shapes within the microstructure of tool steel [98]. 

Common morphologies of M_6_C carbides include spider-web, fishbone, and plate-like structures, which are formed due to their high iron (Fe), tungsten (W), and molybdenum (Mo) content. During the sintering process at certain temperatures, the formation of metastable M_2_C carbides is detected through X-ray diffraction (XRD), contributing to the overall microstructure of the tool steel [52]. However, these carbides, although identified by XRD analysis, are not visibly discernible within the microstructure due to their nanometer-scale size constraints. Each type of carbide present in tool steel microstructures fulfills specific roles and contributes distinct characteristics to the material’s properties. MC carbides are renowned for their hardness and wear resistance [99]. These carbides play a crucial role in enhancing the abrasion resistance of tool steel, making it suitable for demanding applications in cutting tools and wear-resistant components [100]. M_6_C carbides contribute to the overall strength and toughness of the material. These carbides improve the mechanical properties of tool steel, particularly its resistance to impact and deformation. In contrast, M_2_C carbides, although metastable and challenging to detect, offer unique benefits to tool steel microstructures [101]. With high percentages of W and Mo and minimal Fe, Cr, and V content, M_2_C carbides enhance the fracture toughness, hardness, and compressive strength of the material [102]. Their presence contributes to the formation of fine carbides, which further improve the hot strength and wear resistance of the tool steel.

Quantification of carbides in tool steel microstructures is essential for several reasons. Firstly, it allows for a detailed understanding of the carbide distribution and composition, enabling precise control over the material’s properties. By quantifying the volume fraction, size, and distribution of carbides, manufacturers can optimize processing parameters to achieve the desired balance of mechanical properties. Additionally, quantification facilitates quality control and ensures consistency in material performance across different batches and production runs [103]. In this study, carbides are identified, classified, and quantified based on the SEM images of the material. By employing image analysis methodologies, carbides quantitatively assess the presence and concentration of different phases within the tool steel matrix by utilizing advanced image analysis techniques of image processing algorithms.

### 5.2. Image-Based Techniques for Carbide Quantification

#### 5.2.1. Overview of Image Analysis Methods

In the realm of image analysis, the quantification of colors within an image plays a pivotal role in various scientific disciplines, ranging from material science to biomedical imaging [104]. The process of color quantification involves discerning and measuring specific color regions within an image, often necessitating the identification of distinct shades such as white, black, dark grey, and light grey. Achieving this level of precision typically entails the application of sophisticated image analysis methods tailored to handle the complexities of color segmentation. One fundamental technique employed in color quantification is thresholding, wherein the image is partitioned into different regions based on pixel intensity values [49,105]. By setting appropriate threshold values, pixels with intensity levels corresponding to the desired colors can be isolated. For instance, pixels with high intensity values may denote white regions, while those with low intensity values could represent black areas. Moreover, thresholding can be adjusted to capture nuances in color, enabling the segmentation of various shades of grey [106]. The quantification of carbides was executed by counting the number of pixels within each segmented region and calculating their respective percentages relative to the total pixel count. This quantitative analysis provided valuable insights into the prevalence and distribution of carbide phases within the microstructure. Each pixel in the grayscale image was assigned a numerical value ranging from 0 (representing black) to 255 (representing white). The colors corresponding to carbide phases were identified and quantified through systematic grouping based on intensity contrast. This methodology enabled us to effectively characterize the carbide network connectivity within the M2 tool steel structures, facilitating comprehensive analysis and interpretation of image data in scientific contexts. 

**Figure 13:**
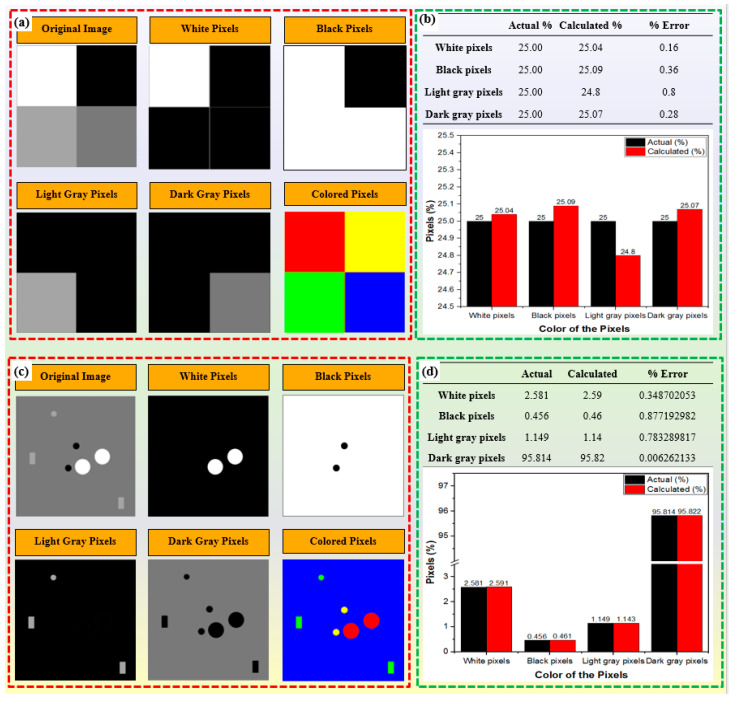
Illustrates the image analysis methodology employed to validate the accuracy of the outcomes, (**a**,**c**) depicting the segmentation of various shapes within the image into four discrete color categories and their corresponding identified output, and (**b**,**d**) showcasing the comparison between the actual and computed percentages of color pixel density.

In this study, color thresholding techniques were applied to quantify carbides within the microstructure. Figure 13 depicts the images utilized to train the model to evaluate the accuracy of carbide quantification. Initially, the model was trained using simple images comprising four distinct color pixels, each occupying 25% of the area, as depicted in Figure 12a. Subsequently, Figure 13b presents a comparison between the model’s computed results and the actual percentages of pixel colors, demonstrating an accuracy of approximately 99.20–99.84%. Another image resembling a microstructure of M2 tool steel was utilized, featuring four distinct colors representing different microstructural components. These include black for pores, white for M_6_C carbides, light gray for MC carbides, and dark gray for the α-Fe matrix. Prior to model execution, all areas were quantified, and the comparison results displayed in Figure 13d reveal an accuracy ranging from 99.13% to 99.99%.

#### 5.2.2. Image Processing of Microstructural Images (SEM) for Carbide Quantification

**Figure 14:**
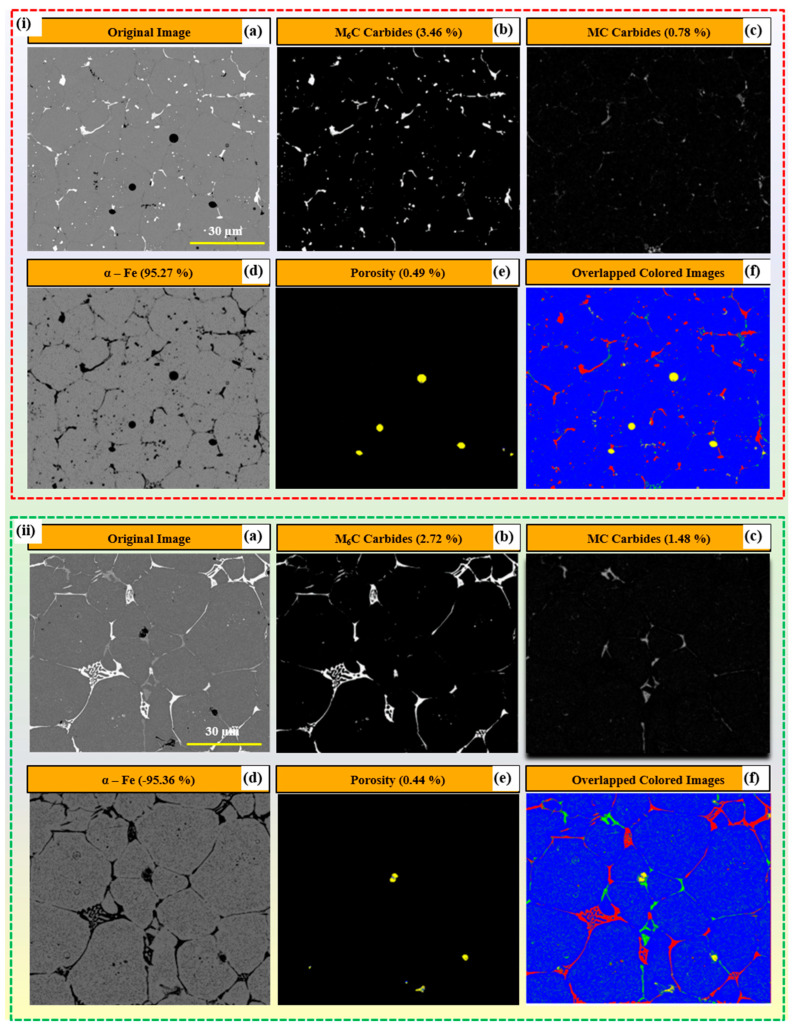
Illustration of the application of image analysis to quantify carbides (M_6_C, MC, α-Fe, and black) within the microstructure of AISI M2 tool steel (**i**) showcasing a sample sintered at 1270 °C for 120 min, followed by furnace cooling, and (**ii**) displaying a sample sintered at 1300 °C for 120 min, followed by furnace cooling.

**Figure 15:**
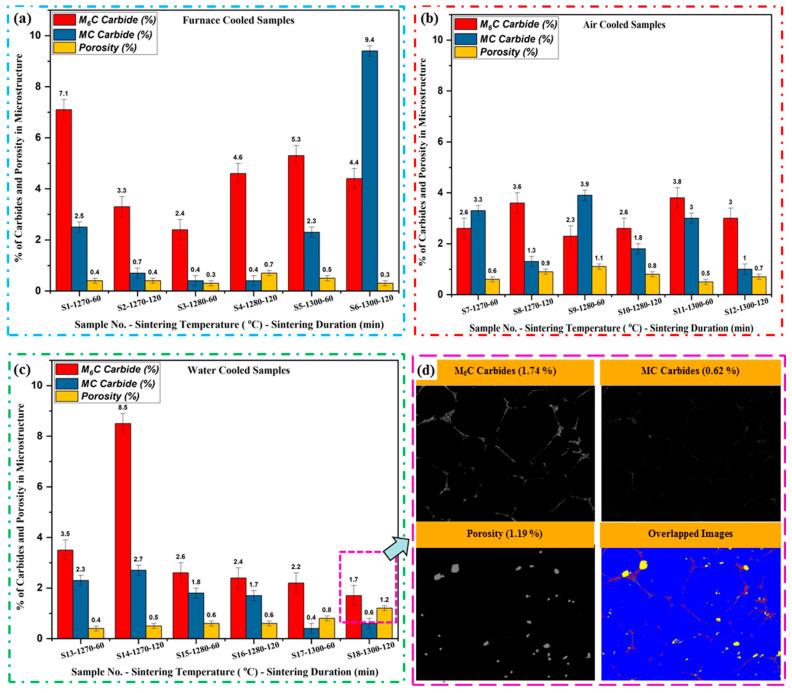
Illustration of the quantification of carbides within the microstructure of AISI M2 tool steel, for (**a**) samples subjected to furnace cooling, (**b**) those cooled with air, and (**c**) samples cooled with water, (**d**) providing an example of sample S18, highlighting the segregation of carbides in the microstructure.

Figure 14a depicts the SEM image segmentation of carbides for sample S2, sintered at 1270 °C for 120 min, followed by furnace cooling. The analysis reveals the presence of M_6_C carbides, constituting 3.46% (Figure 14i(b)), MC carbides at 0.78% (Figure 14i(c)), porosity at 0.49% (Figure 14i(e)), and α-Fe content within the balanced range (Figure 14i(d)). Multiple (four) images were captured at different locations within the same sample, and the average values were computed to provide quantified results of carbides, as demonstrated in the bar graph of Figure 15. Similarly, for sample S6, sintered at 1300 °C for 120 min, followed by furnace cooling, its microstructure was analyzed. The quantification revealed M_6_C carbides at 2.72% (Figure 14ii(b)), MC carbides at 1.48% (Figure 14ii(c)), porosity at 0.44% (Figure 14ii(e)), and α-Fe content within the balanced range (Figure 14ii(d)). Similar processing was also applied to all the other samples (S2 to S17) to quantify the carbides present in the microstructure of the AISI tool steel. The study investigates the microstructural characteristics of AISI M2 tool steel under different cooling conditions, focusing on the quantification of M_6_C carbides, MC carbides, and porosity percentages using advanced image analysis techniques. Three cooling conditions, namely furnace-cooled, air-cooled, and water-cooled, are examined to assess their influence on the microstructural features of the material. The quantification is performed using SEM images obtained from multiple samples subjected to each cooling condition, providing a comprehensive understanding of the carbide distribution and porosity levels within the microstructure. Figure 15a shows the analysis of carbides and porosity percentage distribution of the furnace-cooled samples. The predominant presence of M_6_C carbides is observed across all samples, with percentages ranging from 2.4 ± 0.4% to 7.1 ± 0.4%, while MC carbides exhibit relatively lower percentages, ranging from 0.4 ± 0.1% to 2.5 ± 0.1%, except Sample S6. Porosity levels remain relatively low, with values ranging from 0.3 ± 0.2% to 0.7 ± 0.2%, indicating good overall material densification under this cooling condition. These quantified porosities are for the imaged area (~350 × 350 microns), but the volumetric density of the part varies from 93 ± 0.5 to 96 ± 0.5%. In the case of Sample S6, which was sintered at 1300 °C for 120 min duration, the MC carbides (~9.4% ± 0.2) are observed to be more dominant at the grain boundaries than M_6_C (~4.4 ± 0.2%) carbides because of the higher sintering duration, which reassembled the carbides at the grain boundaries during the grain growth process.

In contrast, samples subjected to air-cooled conditions exhibit lower variability in carbide and porosity percentages, as shown in Figure 15b. MC carbides and M_6_C carbides demonstrate shorter distributions compared to furnace-cooled samples, with percentages varying from 1 ± 0.2% to 3.9 ± 0.2% and 2.3 ± 0.4% to 3.6 ± 0.4%, respectively. Porosity levels also show slight increases compared to furnace-cooled samples (because of oxidation), ranging from 0.5 ± 0.1% to 1.1 ± 0.4%, suggesting differences in cooling rates and microstructural evolution under this condition. In all air-cooled samples except for S7 and S9, the M_6_C carbide predominates over the MC carbide, as shown in Figure 15b.

Water-cooled samples further have the higher variability in MC and M_6_C carbide percentages spanning from 0.41 ± 0.2% to 2.73 ± 0.2% and 1.71 ± 0.4% to 8.52 ± 0.4%, respectively. Porosity levels show a slight increase compared to air-cooled samples, with values ranging from 0.4 ± 0.1% to 1.2 ± 0.1% due to oxidation. The higher percentage of M_6_C carbides in certain water-cooled samples suggests potential differences in carbide precipitation kinetics under this cooling condition.

Overall, the study underscores the influence of cooling conditions on the microstructural characteristics of AISI M2 tool steel, as evidenced by variations in carbide distribution and porosity levels. These findings provide valuable insights for process optimization and quality control in AM and metallurgical applications, contributing to the advancement of materials science and engineering. 

Defect analysis and carbide quantification of microstructure in AM present several challenges, primarily related to sensitivity, complexity, and computational demands. A major limitation is the variable sensitivity to different defect types, where smaller or low-contrast anomalies can escape detection, leading to underestimation. Carbide quantification is susceptible to errors stemming from image noise, inconsistent contrast, and resolution constraints. The proposed defect analysis and quantification methods, despite their high accuracy, face inherent limitations and challenges that could affect their reliability. The reliance on grayscale image processing and thresholding techniques for segmentation is sensitive to variations in image quality, contrast, and lighting, potentially leading to inconsistent detection of microstructural features. This sensitivity could result in misclassification or omission of smaller or low-contrast defects, such as micro-cracks or tiny pores, thereby impacting the thoroughness of defect analysis. Errors in carbide quantification can arise from these segmentation issues, compounded by the complexity of real-world microstructures where overlapping or indistinct features complicate accurate thresholding. Additionally, the computational demands of high-resolution image processing may limit scalability, posing challenges for large-scale or real-time applications. These factors suggest the need for more robust methodologies and enhanced model training to improve sensitivity and generalization in diverse microstructural contexts.

Future research in defect analysis for BJ-AM could focus on integrating camera-based systems to continuously monitor and analyze each layer during the printing process. This advanced imaging technique would capture high-resolution images in real time, allowing for immediate identification of anomalies or defect formation at the layer-by-layer level. Using sophisticated image analysis algorithms, the system could detect early signs of defects, such as porosity, layer misalignment, or irregular powder distribution. If potential defects are detected, the system could automatically adjust process parameters, such as binder flow rate, layer thickness, or printing speed, to mitigate or prevent defect formation. By implementing this real-time monitoring approach, the binder jetting process could become more adaptive, reducing the incidence of defects in the final printed parts. This proactive defect minimization technique could significantly enhance the quality and reliability of binder jet components, offering a robust solution for additive manufacturing quality control.

## 6. Conclusions

In conclusion, this research contributes significantly to the advancement of quality control practices in binder jetting additive manufacturing (BJ-AM). By delving into the binding mechanisms and conducting comprehensive defect analysis, the research underscores the critical importance of defect (in-process, post-process, and sintering) identification for quality control in BJ-AM. This study also offers a systematic and rigorous scientific approach to meticulously analyze the defects encountered during the printing process provides insights into their root causes and proposes effective strategies to mitigate their occurrence. These proactive measures not only prevent catastrophic defect formation in the sintered parts but also contribute to the overall improvement in part quality. Moreover, the study delves into the critical concern of shrinkage in sintered parts, a prevalent issue in BJ-AM. It was noted that furnace-cooled samples exhibited the lowest shrinkage rates (ranging from approximately 1.7 ± 0.4% to 5 ± 0.4%), followed by air-cooled samples (ranging from 1.89 ± 0.4% to 8 ± 0.4%), while water-cooled samples showed the highest shrinkage rates (ranging from 2 ± 0.4% to 14.1 ± 0.4%).

In addition to defect analysis and remediation, this research underscores the significance of quantifying carbides within the microstructure of AISI M2 tool steel, thereby providing a comprehensive understanding of material properties. By elucidating the importance of carbide quantification, the study demonstrates how SEM images can be effectively processed using advanced image analysis techniques to extract valuable information. It is observed that the M2 tool steel consists of a high percentage of M_6_C carbides as compared to MC at most of the sintering conditions. In furnace-cooled samples, the detected M_6_C carbide was very high (~2.4 to 7.1%) with MC (0.4 to 9.4%). In air-cooled samples, there was less variability in M_6_C (2.6 to 3.8%) and MC (0.5 to 1.1) carbides. The highest M_6_C carbide percentage of 8.52% was observed in water-cooled samples. This detailed analysis facilitates the optimization of processing parameters and material composition, ultimately enhancing the quality and performance of BJ-AM components. The future scope encompasses developing a model capable of real-time defect detection during the printing process, with the capacity to autonomously take corrective actions as defects arise. Additionally, there is potential for further investigation into analyzing the average size of carbides present in the microstructure, providing deeper insights into material properties and aiding in process optimization.

## Figures and Tables

**Table 1:** The chemical composition (weight %) of the AISI M2 tool steel powder [51,52].

Powder Size	C	W	Mo	Cr	V	Si	Mn	S	P	Fe
10 microns	0.9–0.95	~5.57	~4.73	~3.95	~1.79	~0.37	~0.27	~0.007	~0.015	Bal.

**Table 2:** Comprehensive overview of the specimens’ progression through the curing, debinding, and sintering stages, followed by cooling via furnace, air, and water methods.

Sample Number	Curing Temperature (°C)	Curing Duration (Hrs)	Debinding Temperature (°C)	Debinding Duration (Hrs)	Sintering Temperature (°C)	Sintering Duration (Minutes)	Types of Cooling
S1	200	14	480	1	1270	60	FC
S2	200	14	480	1	1270	60	FC
S3	200	14	480	1	1280	60	FC
S4	200	14	480	1	1280	60	FC
S5	200	14	480	1	1300	60	FC
S6	200	14	480	1	1300	60	FC
S7	200	14	480	1	1270	60	AC
S8	200	14	480	1	1270	60	AC
S9	200	14	480	1	1280	60	AC
S10	200	14	480	1	1280	60	AC
S11	200	14	480	1	1300	60	AC
S12	200	14	480	1	1300	60	AC
S13	200	14	480	1	1270	60	WC
S14	200	14	480	1	1270	60	WC
S15	200	14	480	1	1280	60	WC
S16	200	14	480	1	1280	60	WC
S17	200	14	480	1	1300	60	WC
S18	200	14	480	1	1300	60	WC

Note: FC: Furnace-Cooled; AC: Air-Cooled and WC; Water-Cooled.

## Data Availability

The data presented in this study are available on request from the corresponding author.
